# Supplementary material for: Incidence, Predictors, and Management of Femoral Vascular Complications Following Catheter Ablation for Atrial Fibrillation: A Systematic Duplex Ultrasound Study
Source: Biomedicines. 2025 Jan 28;13(2):314. doi: 10.3390/biomedicines13020314 (PMC11853090; doi:10.3390/biomedicines13020314)
Supplement: Supplementary file 1 [file biomedicines-13-00314-s001.zip › biomedicines-3433867-supplementary.pdf]

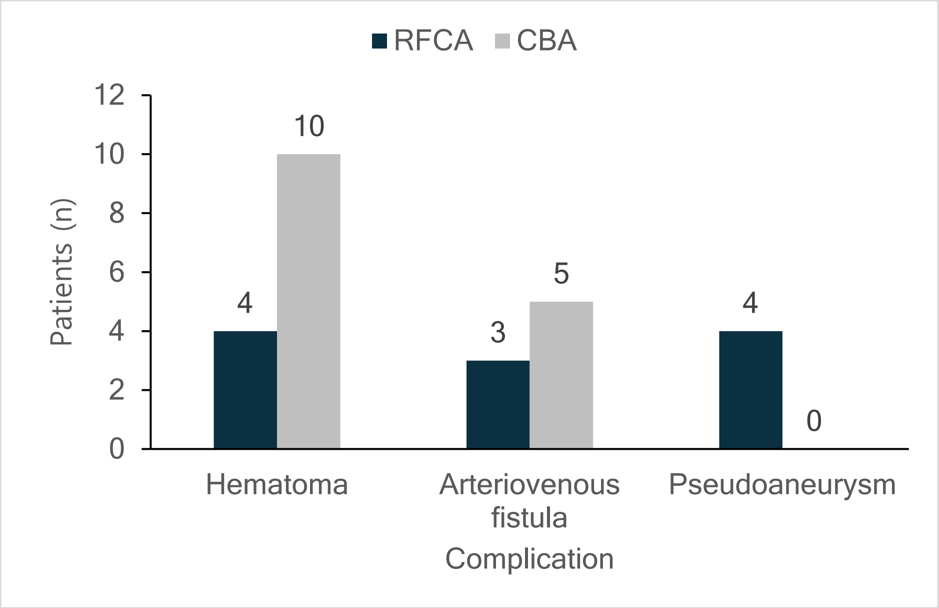

	Hematoma	Arteriovenous fistula	Pseudoaneurysm
RFCA	4 (2.2%)	3 (1.6%)	4 (2.2%)
CBA	10 (4.5%)	5 (2.3%)	0 (0%)

Supplementary Figure S1. Comparison of vascular complications between patients who underwent radiofrequency ablation and cryoballoon ablation. CBA, cryoballoon ablation; RFCA, radiofrequency ablation.
